# Loss of appetite in patients with amyotrophic lateral sclerosis is associated with weight loss and anxiety/depression

**DOI:** 10.1038/s41598-021-88755-x

**Published:** 2021-04-27

**Authors:** Yajun Wang, Shan Ye, Lu Chen, Lu Tang, Dongsheng Fan

**Affiliations:** 1 Department of Neurology, Peking University Third Hospital, Beijing, 100191 China; 2 Beijing Municipal Key Laboratory of Biomarker and Translational Research in Neurodegenerative Diseases, Beijing, China

**Keywords:** Neurology, Risk factors

## Abstract

Weight loss is common in patients with Amyotrophic lateral sclerosis (ALS), and associated with disease progression. Loss of appetite has been shown to be a contributor to weight loss in patients with amyotrophic lateral sclerosis (ALS). However, the reason of loss of appetite is not clear. The Council on Nutrition appetite questionnaire (CNAQ) and the simplified nutritional appetite questionnaire (SNAQ) are short and simple appetite assessment tools, which were using in ALS patients. In our study, the CNAQ and SNAQ were translated into Chinese, and their reliability and validity were tested. The Chinese version of the CNAQ (CNAQ-C) presented more appropriate reliability and validity than the SNAQ. Among the 94 ALS patients, 50 patients (53.2%) had loss of appetite, and we found that anxiety and/or depression contributed to the loss of appetite in the ALS patients. We reconfirmed that loss of appetite was associated with greater weight loss but not with clinical features of ALS. The loss of appetite caused by emotional problems in ALS patients should be taken seriously, and early intervention should be implemented to reduce weight loss.

## Introduction

Amyotrophic lateral sclerosis (ALS) is a clinically and genetically heterogeneous, multidomain neurodegenerative disease characterized by degeneration of motor neurons in the brain and spinal cord^1,2^. ALS usually begins with focal weakness, as in limb or bulbar regions, and then expands to involve most muscles, including the diaphragm. Eventually, patients die of respiratory paralysis^3^.

Weight loss is common in patients with ALS. In fact, the correlation between body mass index (BMI) and ALS disease progression has been well confirmed^4–6^. Two-thirds of patients with ALS have weight loss at diagnosis, the rate of weight loss from onset to diagnosis has been found to be an independent prognostic factor in ALS^4^, and patients with higher BMI at the time of first visit had a longer survival^5^. Moreover, body weight change after diagnosis is predictive of survival in ALS, and weight gain after diagnosis improves survival prognosis^6^. Furthermore, we have recently shown that life course adiposity, taken as a whole, could reduce the risk of ALS^7^. The causes of weight loss in ALS are multifactorial and overlapping and may be related to dyspnoea^8^, dysphagia^9^, etc. Loss of appetite has been shown to be associated with weight loss, reduction in BMI, and loss of fat mass in ALS patients^10^. Approximately 18–47% of ALS patients suffer from anorexia, and this percentage increases as the disease progresses^8,10,11^. However, the cause for loss of appetite in ALS patients is not clear and may be related to dyspnoea^8^, although this has not been supported by other research. In contrast, other studies have shown that loss of appetite has no correlation with clinical measurements, such as respiratory function and bulbar function^10,11^.

The Council on Nutrition appetite questionnaire (CNAQ) and the simplified nutritional appetite questionnaire (SNAQ) are short and simple appetite assessment tools developed by Wilson et al. in 2005 to predict weight loss in community-dwelling adults and long-term care residents^12^. To date, the CNAQ and SNAQ have been translated into multiple languages^13–15^ and used for detecting the appetite of patients with a variety of diseases^16–18^, including ALS^8,10,11^. However, there are currently no Chinese versions of the CNAQ and SNAQ. Therefore, our research had the following aims: (1) we translated the CNAQ and SNAQ into Chinese and tested their reliability and validity in ALS patients and (2) analysed the possible risk factors for the loss of appetite in ALS patients.

## Results

### Characteristics of patients

A total of 94 patients were enrolled in this study, including 64 males with a mean age of 51.72 (95% confidence interval (CI), 48.75–54.69) and 30 females with a mean age of 52.03 (95% CI 48.14–55.92). In total, 37% of the patients had a history of smoking, and 24% of the patients had a history of long-term alcohol consumption. The median score of the ALSFRS-R, bulbar subscore (questions 1 to 3) and respiratory subscore (question 10) were 39.5 (Interquartile range (IQR), 11), 11.50 (IQR, 2) and 12.00 (IQR, 2), respectively. For these patients, the mean weight at screening was 66.26 (95% CI 63.97–68.55), and the mean BMI at screening was 23.72 (95% CI 23.03–24.42). The median duration of disease and diagnostic delay were 21.00 months (IQR, 14.75) and 13.83 (IQR, 13.20), respectively. Fifteen of 94 ALS patients were bulbar onset. The baseline characteristics of the patients are presented in Table 1.

**Table 1:** Characteristics of ALS patients at the time of collection of baseline data.

Characteristic	Total	Male	Female
n (%)	94 (100)	64 (68.1)	30 (31.9)
Age, year [mean (95% CI)]	51.82 (49.49–54.15)	51.72 (48.75–54.69)	52.03 (48.14–55.92)
Drinking, n (%)	24 (25.5)	21 (32.8)	3 (10.0)
Smoking, n (%)	37 (39.4)	34 (53.1)	3 (10.0)
ALSFRS-R score [median (IQR)]	39.50 (11.00)	39.00 (11.00)	40.00 (10.00)
ALSFRS-R, Bulbar sub-scores [median (IQR)]	11.50 (2.00)	12.00 (2.00)	10.00 (4.00)
ALSFRS-R, Resp sub-scores [median (IQR)]	12.00 (2.00)	12.00 (2.00)	11.00 (2.00)
Weight at screening [mean (95% CI)]	66.26 (63.97–68.55)	69.76 (67.10–72.41)	58.78 (55.65–61.92)
BMI at screening [mean (95% CI)]	23.72 (23.03–24.42)	24.09 (23.23–24.96)	22.94 (21.79–24.09)
Duration of disease, months [median (IQR)]	21.00 (14.75)	22.00 (13.50)	20.00 (17.75)
Diagnostic delay, months [median (IQR)]	13.83 (13.20)	12.37 (13.68)	15.92 (13.73)
Bulbar onset, n (%)	15 (16)	7 (10.9)	8 (26.7)

*CI* confidence interval, *IQR* interquartile range, *BMI* body mass index, *ALS* amyotrophic lateral sclerosis, *ALSFRS-R* ALS Functional Rating Scale-Revised.

### Reliability/validity of the CNAQ-C and SNAQ-C

We collected information from 94 patients to analyse the reliability and validity of the CNAQ-C and SNAQ-C. Cronbach’s α coefficients for the CNAQ-C and SNAQ-C were 0.667 and 0.662, respectively. The CFIs for the CNAQ-C and SNAQ-C were all > 0.9, and the RMRs were < 0.01. The GFIs were all > 0.9, and the AGFIs were all > 0.8 for the CNAQ-C and SNAQ-C. The χ^2^/df and RMSEA for the CNAQ-C were 1.35 and 0.058, respectively, and the χ^2^/df and RMSEA for the SNAQ-C were 3.03 and 0.14, respectively, indicating that the CNAQ-C exhibited a better fit to the model using confirmatory factor analysis (Table 2). Therefore, we used the CNAQ-C for subsequent analyses.

**Table 2:** Comparison of values for fitting to the structural equation model.

	χ^2^/df	GFI	CFI	RMR	AGFI	RMSEA
CNAQ-C	1.35	0.94	0.96	0.029	0.89	0.058
SNAQ-C	3.03	0.97	0.97	0.014	0.86	0.14

*RMR* root of the mean square residual, *CFI* comparative fit index, *GFI* goodness of fit index, *AGFI* adjusted goodness of fit index, *RMSEA* root mean square error of approximation, *CNAQ-C* Council on Nutrition Appetite Questionnaire- Chinese version, *SNAQ-C* simplified Nutrition Appetite Questionnaire- Chinese version.

### Comparison between ALS patients with intact appetite and loss of appetite

Among the 94 ALS patients, 44 patients (46.8%) had intact appetite, and 50 patients (53.2%) had loss of appetite. Age (p = 0.958), sex (p = 0.671) and the percentage of smoking and alcohol abuse (p = 0.893; p = 0.190) were no significant differences between ALS patients with intact appetite and loss of appetite. The weight and BMI at screening of ALS patients with intact appetite was higher than that of ALS patients with loss of appetite (p = 0.015; p = 0.005). There was no difference in other clinical measurements, including ALSFRS-R scores (p = 0.227), bulbar subscore (p = 0.690), respiratory subscore (p = 0.712), duration of disease (p = 0.943), diagnostic delay (p = 0.570) and the percentage of bulbar onset (p = 0.254) between the two groups (Table 3).

**Table 3:** Characteristics of ALS patients with intact appetite (CNAQ > 28) and loss of appetite (CNAQ ≤ 28) at the time of baseline.

Characteristic	CNAQ > 28(n = 44)	CNAQ ≤ 28(n = 50)		P value
Age, year [mean (95% CI)]	51.9 (48.73–55.05)		51.8 (48.27–55.25)	0.958
Sex (female), n (%)	15 (34.1)		15 (30)	0.671
Drinking, n (%)	14 (31.82)		10 (20)	0.190
Smoking, n (%)	17 (38.64)		20 (40)	0.893
ALSFRS-R score [median (IQR)]	40.00 (10.00)		39.00 (9.50)	0.227
ALSFRS-R, Bulbar sub-scores [median (IQR)]	11.50 (2.00)		11.50 (3.00)	0.690
ALSFRS-R, Resp sub-scores [median (IQR)]	12.0 (1.00)		12.00 (2.00)	0.712
Weight at screening [median (IQR)]	70 (18.75)		63.50 (15.00)	0.015*
BMI at screening [median (IQR)]	25.71 (4.83)		22.64 (4.89)	0.005**
Duration of disease, months [median (IQR)]	21.00 (21.25)		21.00 (11.50)	0.943
Diagnosis delay, months [median (IQR)]	13.05 (16.31)		14.45 (11.58)	0.570
Bulbar onset, n (%)	5 (11.36)		10 (20)	0.254
HADS-A score [median (IQR)]	5.00 (4.00)		7.00 (6.25)	0.001**
HADS-D score [mean (95% CI)]	5.14 (4.09–6.18)		8.42 (7.38–9.46)	< 0.001***

*CI* confidence interval, *IQR* interquartile range, *BMI* body mass index, *ALS* amyotrophic lateral sclerosis, *ALSFRS-R* ALS Functional Rating Scale-Revised, *HADS-A* Hospital Anxiety and Depression Scale-Axiety, *HADS-D* Hospital Anxiety and Depression Scale-Depression. *p < 0.05; **p < 0.01; ***p < 0.001.

**Figure 1:**
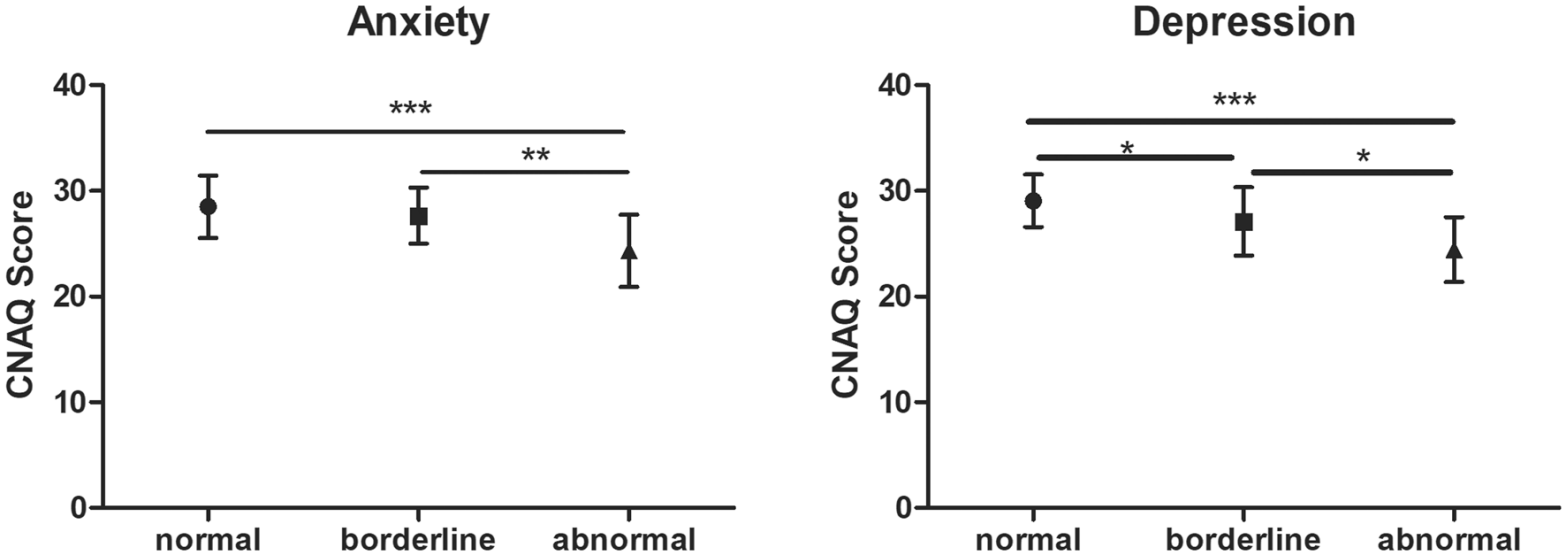
According to the score of HADS-A and HADS-D, separated the patients into normal, borderline and abnormal groups, CNAQ scores of normal, borderline and abnormal groups were compared. *p < 0.05; **p < 0.01; ***p < 0.001.

Among the 94 ALS patients, 52% of patients had anxiety and/or depression. In our results, the HADS-A and HADS-D scores of the ALS patients with intact appetite were lower than those of the ALS patients with loss of appetite (p = 0.01; p < 0.001) (Table 3), indicating that the emotional state of patients in the normal appetite group was better than that in the loss of appetite group. Moreover, according to the HADS-A and HADS-D scores, we separated the patients into normal, borderline and abnormal groups; the CNAQ-C scores of the borderline and abnormal patients were lower than those of the normal patients (p < 0.001) (Fig. 1), indicating that anxiety and depression might be potential factors associated with the loss of appetite.

To assess the cognitive and behavioural status of ALS patients, the ECAS was used in our research. Eighty-one of 94 participants completed the ECAS, and the scores of 15 patients less than 81. For these patients, 40 patients (49.4%) had intact appetite, and 41 patients (50.6%) had loss of appetite. Surprisingly, there were no significant differences in the ECAS scores (p = 0.688), the ALS-special function and ALS-non-special function scores (p = 0.839; p = 0.917) between the two groups (Supplementary Table S1).

### Correlations between CNAQ-C and baseline disease characteristics

Correlation analysis showed that CNAQ-C scores was correlated with weight and BMI since diagnosis (r = − 0.29, p = 0.006; r = − 0.277, p = 0.007). The CNAQ-C scores and ALSFRS-R scores exhibited a low correlation, despite the p-value was close to 0.05 (r = 0.207, p = 0.046). However, no obvious relationship was found between CNAQ-C scores and bulbar sub-scores (r = 0.172, p = 0.098), and respiratory sub-scores (r = 0.145, p = 0.145). There was also no correlation between the CNAQ score and duration of disease (r = − 0.303, p = 0.772), and diagnosis delay (r = 0.060, p = 0.565). HADS-A and HADS-D had correlation with CNAQ-C scores (r = − 0.432, p < 0.001; r = − 0.493, p < 0.001) (Table 4). For the ECAS, the results showed that CNAQ-C scores have no correlation with total ECAS (r = 0.111, p = 0.326), ALS-special function (r = 0.016, p = 0.154), ALS-non-special function scores (r = 0.025, p = 0.822) (Supplementary Table S2).

**Table 4:** Correlations between CNAQ scores and baseline disease characteristics.

Characteristic	n	Correlation coefficient	P value
Weight loss since diagnose	94	− 0.279	0.006**
BMI decrease since diagnose	94	− 0.277	0.007**
ALSFRS-R	94	0.207	0.046*
ALSFRS-R, Bulbar sub-scores	94	0.172	0.098
ALSFRS-R, Resp sub-scores	94	0.090	0.386
Duration of disease, months	94	− 0.030	0.772
Diagnosis delay, months	94	− 0.060	0.565
HADS-A score	94	− 0.432	< 0.001***
HADS-D score	94	− 0.493	< 0.001***

*ALS* amyotrophic lateral sclerosis, *ALSFRS-R* ALS Functional Rating Scale-Revised, *BMI* body mass index, *HADS-A* Hospital Anxiety and Depression Scale-Axiety, *HADS-D* Hospital Anxiety and Depression Scale-Depression. *p < 0.05; **p < 0.01; ***p < 0.001.

To determine whether the relationships between CNAQ-C and HADS-A, and HADS-D were independent of other clinical features, multivariate stepwise regression analysis was performed with CNAQ-C as dependent variable. Univariate correlation analysis showed that CNAQ-C scores were associated with ALSFRS-R score (r = 0.258, p = 0.012), Weight at screening (r = 0.335, p = 0.001), BMI at screening (r = 0.373, p < 0.001), HADS-A score (r = 0.467, p < 0.001), HADS-D score (r = 0.511, p < 0.001). Multivariate stepwise regression analysis showed that HADS-D (B = − 0.457, p < 0.001) was independently associated with CNAQ-C adjust for ALSFRS-R score, Weight at screening, BMI at screening, HADS-A score (Table 5).

**Table 5:** Multivariable linear regression model of CNAQ-C.

Independent variable	Beta coefficient	Standard error	t	P value
ALSFRS-R score	0.173	0.038	2.007	0.048*
BMI at screening	0.249	0.086	2.842	0.006**
HADS-D score	− 0.457	0.072	− 5.381	< 0.001***

*ALSFRS-R* ALS Functional Rating Scale-Revised, *BMI* body mass index, *HADS-D* Hospital Anxiety and Depression Scale-Depression. *p < 0.05; **p < 0.01; ***p < 0.001.

## Discussion

We translated and back-translated the CNAQ and its simplified version the SNAQ into Chinese (the CNAQ-C and SNAQ-C, respectively), and reliability and validity were tested. We found that the Chinese version of the CNAQ had sufficient reliability and validity. We showed that approximately half of the ALS patients had loss of appetite, which was partly due to anxiety and depression. Our data supported the notion that weight loss was associated with the loss of appetite and reconfirm the view that loss of appetite is a potential contributor to weight loss in ALS patients.

Epidemiological evidence shows that ALS patients begin to lose weight preceding motor symptoms by several years^19^. Furthermore, patients with greater weight loss tend to have worse ALSFRS-R scores and shorter survival times^4,20^. There are many factors that contribute to weight loss with ALS, and loss of appetite has proven to be a potential contributor^8,10,11^. The CNAQ and SNAQ were developed to predict weight loss in community-dwelling adults and long-term care residents and have been used to detect the appetite of ALS patients^8,10,11^. In our research, we developed the Chinese versions of the CNAQ and SNAQ. The Cronbach’s α coefficients of the CNAQ-C and SNAQ-C were 0.667 and 0.662, respectively, which were slightly lower than 0.7. In the study by Wilson et al.^12^, Cronbach’s α coefficients for the CNAQ and SNAQ were 0.470 and 0.510 in the long-term care group and 0.72 and 0.70 in the community-dwelling group, respectively. This may be due to the different subjects assessed in the two studies. In addition, the CNAQ-C presented good fit in the confirmatory factor analysis as assessed by multiple indices, while some indices with the SNAQ-C were not up to standard, indicating that the CNAQ-C is more suitable for our research.

Approximately half of the participants (53.2%) demonstrated severe loss of appetite in our study, which was similar to the percentage in Holm et al. (47%)^12^ and was higher than that in Ngo et al. (29%)^10^ and Mezoian BA et al. (18%)^11^. The underlying causes for loss of appetite in ALS patients are unclear. Holm et al. found a significant association between dyspnoea and loss of appetite, while Ngo et al. and Taylor Mezoian BA et al. did not confirm this finding. Likewise, our study also found no significant differences in bulbar and respiratory scores between ALS patients with intact appetite vs. ALS patients with loss of appetite. However, the respiratory scores of ALSFRS-R provided limited information about the actual respiratory status. In future research, more sensitive tests, as forced vital capacity, nocturnal oximetry, supine spirometry, etc. should be conducted.

Anxiety and depression are common in ALS patients. Nimish J Thakore et al. reported that 33% of patients had at least moderate depression^21^. The prevalence of anxiety in ALS patients ranged from 0 to 30%^22^. Patients with depression showed significant heterogeneity in appetite, with approximately 48% of adults with depression showing depression-related loss of appetite and approximately 35% showing depression-related increases in appetite^23^. Anxiety and depression have been confirmed to be associated with changes in appetite in a variety of diseases^24,25^. In our study, anxiety and depression showed significant differences between ALS patients with intact appetite vs. ALS patients with loss of appetite, and the CNAQ-C score in the anxiety and depression group was significantly lower than that in the normal group, which had not been mentioned in previous studies, as no professional psychological scales were used. Multivariate stepwise regression analysis showed that HADS-D was independently associated with CNAQ-C, indicating that depression might be an independent correlative factor for the loss of appetite in ALS patients. Although there is not enough evidence to show that ALS patients with loss of appetite could be improved by psychological regulation, consider emotional problems and give appropriate treatment should be reasonable.

The view that the motivation to eat depends on cognitive regulation of reward processes is gaining support, with control of appetite thought to involve cognitive processes such as learning, attention and memory^26^. These cognitive processes may be engaged during various aspects of appetite control, including before, during and between meals^27^. Among a sample of Chinese ALS patients, 35.71% showed cognitive impairment, and 27.38% showed behavioural abnormalities^28^. In our study, however, no association was found between ECAS and CNAQ scores. which might be due to few patients had an ECAS score below 81 in our research (only 15 patients). Interestingly, previous study has shown that, compared with ALS patients, patients with behavioural variant frontotemporal dementia (bvFTD) were more likely to show an increase in appetite^29^, which was verified that cognitive impairment had an impact on the appetite of ALS patients. Therefore, we believe that ECAS should be used as a screening indicator to evaluate cognitive and behavioral changes of ALS patients in subsequent appetite studies.

In our study, 52% of patients had anxiety and/or depression. Interestingly, we found that after removing the patients with anxiety and/or depression, still had sixteen patients (approximately 33%) with loss of appetite, which shows that emotion dysfunction was not the only reason for the loss of appetite in ALS patients. Unexpectedly, similar to previous results, the weight and BMI at the screening of ALS patients with intact appetite were higher than those of ALS patients with loss of appetite, and there were no differences in other demographic data and clinical measurements between the two groups (Supplementary Table S3). Likewise, no significant differences were found in the mean ECAS, ALS-special function and ALS-non-special function scores between ALS patients with intact appetite and ALS patients with loss of appetite (Supplementary Table S4). Similarly, the CNAQ score has no correlation with other clinical indicators except the weight loss and BMI decrease since diagnosis (Supplementary Table S5). The disruption of central energy homeostasis may play an important role. The hypothalamus, the main central organ that regulates appetite, has been shown to atrophy in ALS patients, even in the premorbid stage, and the degree of atrophy was correlated with BMI^30^. Moreover, changes in appetite-regulating AgRP (increased) and POMC (decreased) neurons have also been demonstrated in ALS mouse model^31^. The volume of multiple brain regions involved in appetite regulation has also been reported to be reduced in ALS patients^32^. In future studies, the effect of central organ alterations on the appetite of ALS patients should be considered.

Our research has the following limitations: (1) We did not collect CNAQ-C information in a control group to compare with the appetite of ALS patients, and we lacked an assessment of energy intake. (2) This study did not have follow-up data. Although the study confirmed the relationship between weight loss and appetite loss, it did not confirm that the CNAQ-C can predict weight loss in the next 6 months. In addition to confirming the functional predictions of the CNAQ, it is necessary to clarify whether there is a relationship between the patient's appetite and disease progression and survival. In conclusion, we identified a new risk factor for loss of appetite in ALS patients, and we emphasized the possibility of other mechanisms. Identification of the mechanisms underlying for the loss of appetite in ALS patients, such as changes in the central nervous system, hormones, and mood, can lead to implementing targeted treatments or using appetite-enhancing drugs based on the mechanisms to prevent patients from losing weight.

## Methods

### Participants

Patients with possible, probable, or definite ALS were included in the study, and all patients met the revised E1 Escorial criteria for ALS from Peking University Third Hospital. The exclusion criteria included digestive system diseases, thyroid diseases, diabetes and other wasting diseases; gastrostomy or nasal feeding and other patients unable to eat; and/or a history of other neurological disorders. A total of 94 patients were enrolled. All participants underwent clinical testing and epidemiological investigations at screening, such as age, weight, BMI, ALS Functional Rating Scale-Revised (ALSFRS-R) scores, duration of disease, diagnosis delay. Smoking was defined as having smoked at least 1 cigarette a day for at least 1 year or more than 360 cigarettes in total for a year. Drinking was defined as having an average of 2 or more drinks per week for more than 1 year. This study was approved by the Research Ethics Committee of Peking University Third Hospital. Written informed consent was provided by all participants. All methods were performed in accordance with relevant guidelines and regulations.

### Translation of the CNAQ and SNAQ

The CNAQ is an 8-item questionnaire about participants' appetite, hunger frequency, satiety, taste, eating behaviour, and mood. The score for each question is 1–5 points, the total score range is 8–40 points, and scores ≤ 28 points are considered indicative of loss of appetite^12^. The SNAQ is a simplified version of the questionnaire composed of 4 questions from the CNAQ. Similarly, each item is rated on a 5-point scale, the total score range is 4–20 points, and scores ≤ 14 points are considered indicative of loss of appetite^12^. We obtained permission from the original article’s author John E Morley to develop the Chinese versions of the CNAQ and SNAQ. Using standardized translation and back-translation methods, the Chinese version (CNAQ-C) was developed by a nutrition researcher, a physician, and a neurobiology researcher, repeated translation and back-translation occurred until equivalent English expressions were attained, and they were approved by the original author (Supplementary Table S6). All participants needed to recall their feelings or behaviours from the past month to answer each question.

### Reliability and validity testing

Reliability was assessed using Cronbach’s α coefficient to establish the internal consistency of the CNAQ-C and SNAQ-C. Lisrel 8.80 was used to perform confirmatory factor analysis (CFA) to examine the fit of the model. We report χ^2^/df, root of the mean square residual (RMR), comparative fit index (CFI), goodness of fit index (GFI), adjusted goodness of fit index (AGFI) and root mean square error of approximation (RMSEA). It is generally considered that if χ^2^/df is approximately 1, CFI, GFI and AGFI are close to 1, and RMR and RMSEA are less than 0.1, these values indicate a good fit.

### Edinburgh Cognitive and Behavioural ALS Screen (ECAS)

The Chinese version of the ECAS was used to evaluate the cognitive function of the participants. The ECAS includes ALS-specific functions and non-ALS-specific functions. The cognitive domains of executive function, verbal fluency and language belong to ALS-specific functions, and the cognitive domains of memory and visuospatial functions belong to non-ALS-specific functions. Based on the characteristics of frontotemporal dementia (FTD), the questions in caregiver interviews are concentrated in five behavioural domains and three psychiatric domains.

### Hospital Anxiety and Depression Scale (HADS)

The HADS is composed of the HADS-A and HADS-D, which are used to detect anxiety and depressive states, respectively. Each subscale consists of 7 items, and the score for each item is 0–3 points. The score of each subscale ranges from 0 to 21. A score of 0–7 is considered normal, 8–10 points is considered borderline, and 11–21 points is considered abnormal, meaning that the patients have anxiety or depression. All participants needed to recall their feelings or behaviours from the past month to answer each question.

### Statistical analysis

For statistical analyses, SPSS version 25.0 was used, and p < 0.05 (two-tailed) was assumed to be statistically significant. Kolmogorov–Smirnov test was used for analysing normal distribution. To compare variables such as age, ALSFRS-R score, weight at screening, BMI at screening, duration of disease, diagnosis delay, HADS score, and ECAS score, the two-sided *t* test was used if the variables were normally distributed, if not, the Mann–Whitney *U* test was used. The chi-square or Fisher’s tests were used when comparing categorical variables such as sex, alcohol, smoking, and bulbar onset. Pearson correlation coefficients were used to calculate correlations between CNAQ-C scores and other variables when the data were continuous, normally distributed, otherwise, Spearman correlation analysis was used. Univariate correlation analysis and multivariate stepwise regression analysis were performed with CNAQ-C as the dependent variable and age, ALSFRS-R score, weight at screening, BMI at screening, duration of disease, diagnosis delay, HADS score as the predicting parameters.

## Supplementary Information

Supplementary Tables.

## Data Availability

All data in this study are included in this article.
