# Supplementary material for: Loss of appetite in patients with amyotrophic lateral sclerosis is associated with weight loss and anxiety/depression
Source: Sci Rep. 2021 Apr 27;11:9119. doi: 10.1038/s41598-021-88755-x (PMC8079393; doi:10.1038/s41598-021-88755-x)
Supplement: Supplementary file 1 — Supplementary Tables. [file 41598_2021_88755_MOESM1_ESM.pdf]

Loss of appetite in patients with amyotrophic lateral sclerosis is associated with weight loss and anxiety/depression

Yajun Wang[1,2], Shan Ye[1,2], Lu Chen[1,2], Lu Tang[1,2], Dongsheng Fan[1,2,*]

[1]Department of Neurology, Peking University Third Hospital, Beijing, China.

[2]Beijing Municipal Key Laboratory of Biomarker and Translational Research in Neurodegenerative Diseases, Beijing, China

*Corresponding author: Dr. Dongsheng Fan

E-mail: dsfan2010@aliyun.com

Department of Neurology, Peking University Third Hospital, Beijing 100191, China.

Dr. Yajun Wang, E-mail: bttre_wyj@sina.com

Dr. Shan Ye, E-mail: yeshanbysy@163.com

Dr. Lu Chen, E-mail: woyaoxiakejian@163.com

Dr. Lu Tang, E-mail: tanglu@bjmu.edu.cn

Dr. Dongsheng Fan, E-mail: dsfan2010@aliyun.com

Table S1. ECAS score of ALS patients with intact appetite (CNAQ>28) and loss of appetite (CNAQ≤28) at the time of baseline.

Characteristic	CNAQ>28(n=40)	CNAQ≤28(n=41)	P Value
Total ECAS score [median (IQR)]	101.00(33.00)	97.00(23.00)	0.688
ALS-special functions [mean (95% CI)]	72.60(67.13-78.07)	71.90(67.64-76.17)	0.839
ALS-non-special functions [median (IQR)]	25.00(11.00)	24.00(7.00)	0.917

CI: confidence interval; IQR: interquartile range;

ECAS: The Edinburgh Cognitive and Behavioural ALS Screen;

ALS: amyotrophic lateral sclerosis.

Table S2. Correlations between CNAQ scores and ECAS scores

Characteristic	n	correlation coefficient	P Value
ALS-special functions	81	0.160	0.154
ALS-non-special functions	81	0.025	0.822
Total ECAS score	81	0.111	0.326

ALS: amyotrophic lateral sclerosis; BMI: body mass index; ECAS: The Edinburgh Cognitive and Behavioural ALS Screen.

Table S3. Characteristics of ALS patients (without anxiety and/or expression) with intact appetite (CNAQ>28) and loss of appetite (CNAQ≤28) at the time of baseline.

Characteristic	CNAQ>28(n=33)	CNAQ≤28(n=16)	P Value
Age, year [mean (95% CI)]	51.97(48.01-55.93)	57.31(50.55-64.07)	0.14
Sex, femal, n (%)	10(30.30)	4(25)	0.962
Drinking, n (%)	12(36.4)	2(12.5)	0.162
Smoking, n (%)	12(63.6)	8(50)	0.362
ALSFRS-R score at screening [mean (95% CI)]	38.12(35.29-40.95)	30.06(35.83-42.29)	0.679
ALSFRS-R, Bulbar sub-scores [median (IQR)]	12.00(2.00)	12.00(1.75)	0.450
ALSFRS-R, resp sub-scores [median (IQR)]	12.00(1.00)	12.00(2.00)	0.905
Weight at screening [median (IQR)]	71.00(19.50)	63.00(15.00)	0.020*
BMI at screening [median (IQR)]	26.04(4.82)	22.16(5.58)	0.003**
Duration of disease (months) [median (IQR)]	23.00(24.00)	25.50(25.00)	0.481
Diagnosis delay (months) [median (IQR)]	14.80(18.28)	18.42(20.68)	0.250
Bulbar onset, n (%)	2(6.10)	3(18.8)	0.383

CI: confidence interval; IQR: interquartile range;

ALS: amyotrophic lateral sclerosis; ALSFRS-R: ALS Functional Rating Scale-Revised;

BMI: body mass index. *, $p<0.05$; **, $p<0.01$

Table S4. ECAS score of ALS patients (without anxiety and/or expression) with intact appetite (CNAQ>28) and loss of appetite (CNAQ≤28) at the time of baseline.

Characteristic	CNAQ>28(n=31)	CNAQ≤28(n=13)	P Value
Total ECAS score [mean (95% CI)]	99.16(91.00-107.32)	94.31(84.73-103.88)	0.42
ALS-special functions [median (IQR)]	77.00(18.00)	74.00(23.50)	0.285
ALS-non-special functions [mean (95% CI)]	24.48(21.97-27.00)	23.00(20.88-25.12)	0.35

CI: confidence interval; IQR: interquartile range;

ECAS: The Edinburgh Cognitive and Behavioural ALS Screen;

ALS: amyotrophic lateral sclerosis.

Table S5. Correlations between CNAQ-C scores and baseline disease characteristics

Characteristic	n	correlation coefficient	P Value
Weight loss since diagnose	49	-0.413	0.003**
BMI decrease since diagnose	49	-0.403	0.004**
ALSFRS-R	49	0.005	0.974
ALSFRS-R, Bulbar sub-scores	49	-0.035	0.813
ALSFRS-R, resp sub-scores	49	0.022	0.881
Total ECAS score	44	0.226	0.081
ALS-special functions	44	0.236	0.085
ALS-non-special functions	44	0.196	0.203

ALS: amyotrophic lateral sclerosis;

ALSFRS-R: ALS Functional Rating Scale-Revised;

BMI: body mass index;

ECAS: The Edinburgh Cognitive and Behavioural ALS Screen.

**, $p<0.01$.

Table S6. The back-translation of CNAQ

**Note: Please tick the most appropriate answer. The score of each question corresponds to the metrics: a=1, b=2, c=3, d=4, e=5, and the sum of all the questions is the CNAQ score. The CNAQ score of less than 28 indicates high risks of the examinee losing weight by at least 5% within 6 months.**	
**1. My appetite is ___.**<br>**a. very bad**<br>**b. bad**<br>**c. normal**<br>**d. good**<br>**e. very good**	**2. When I eat, ___.**<br>**a. I feel full after eating very little food.**<br>**b. I feel full after eating 1/3 of the food.**<br>**c. I feel full after eating 1/2 of the food.**<br>**d. I feel full after eating most of the food.**<br>**e. I don't even feel full after eating all the food.**
**3. How often do I feel hungry?**<br>**a. Seldom**<br>**b. Occasionally**<br>**c. Sometimes**<br>**d. Usually**<br>**e. Always**	**4. I think the taste of food is now ___.**<br>**a. very bad**<br>**b. bad**<br>**c. normal**<br>**d. good**<br>**e. very good**
**5. Compared with before, I think the taste of food is ___.**<br>**a. much worse**<br>**b. worse**<br>**c. not changed**<br>**d. better**<br>**e. much better**	**6. Usually, I would like to eat ___ a day.**<br>**a. less than once**<br>**b. once**<br>**c. twice**<br>**d. three times**<br>**e. more than three times**
**7. How often do I feel sick when eating?**<br>**a. Usually**<br>**b. Often**<br>**c. Sometimes**<br>**d. Seldom**<br>**e. Never**	**8. In terms of emotions, I usually feel_____.**<br>**a. very sad**<br>**b. sad**<br>**c. nothing special**<br>**d. glad**<br>**e. very glad**
